# The Effects of Different Motor Teaching Strategies on Learning a Complex Motor Task

**DOI:** 10.3390/s24041231

**Published:** 2024-02-15

**Authors:** Tjasa Kunavar, Marko Jamšek, Edwin Johnatan Avila-Mireles, Elmar Rueckert, Luka Peternel, Jan Babič

**Affiliations:** 1 Laboratory for Neromechanics and Biorobotics, Department of Automatics and Biocybernetics, Jožef Stefan Institute, 1000 Ljubljana, Slovenia; 2 Jožef Stefan International Postgraduate School, Jamova cesta 39, 1000 Ljubljana, Slovenia; 3 Chair of Cyber-Physical-Systems, Montauniversität Leoben, 8700 Leoben, Austria; 4 Department of Cognitive Robotics, Delft University of Technology, 2628 CD Delft, The Netherlands; 5 Faculty of Electrical Engineering, University of Ljubljana, 1000 Ljubljana, Slovenia

**Keywords:** motor learning, skill learning, human–robot interaction, visuomotor perturbation, motor teaching, tracking task, robot tutoring

## Abstract

During the learning of a new sensorimotor task, individuals are usually provided with instructional stimuli and relevant information about the target task. The inclusion of haptic devices in the study of this kind of learning has greatly helped in the understanding of how an individual can improve or acquire new skills. However, the way in which the information and stimuli are delivered has not been extensively explored. We have designed a challenging task with nonintuitive visuomotor perturbation that allows us to apply and compare different motor strategies to study the teaching process and to avoid the interference of previous knowledge present in the naïve subjects. Three subject groups participated in our experiment, where the learning by repetition without assistance, learning by repetition with assistance, and task Segmentation Learning techniques were performed with a haptic robot. Our results show that all the groups were able to successfully complete the task and that the subjects’ performance during training and evaluation was not affected by modifying the teaching strategy. Nevertheless, our results indicate that the presented task design is useful for the study of sensorimotor teaching and that the presented metrics are suitable for exploring the evolution of the accuracy and precision during learning.

## 1. Introduction

In the last few decades, the study of motor learning has led to great advances in the understanding of the skill acquisition process. In general, motor learning is an internal process that consists of developing cognitive structures through information processing. In the most traditional way, when naïve individuals need to acquire a new skill, they have to rely on the ability of expert individuals to transfer the right kind and amount of information necessary for the performance of such a skill, establishing a teaching–learning interaction. The relationship between the expert and the naïve during such an interaction has been studied mainly in pedagogical environments, either in the classroom or in the physical education field [1,2]. Nevertheless, and despite the big efforts from teachers and pedagogues to develop and implement novel and more effective teaching techniques, the sensorimotor skill teaching process has struggled in innovation regarding the methods behind it. However, teachers and pedagogues have established that motor teaching should focus on the instructional stimuli able to produce the desired learning outcomes [3,4].

Given the heuristic nature of motor learning, spontaneous optimal solutions may appear during task exploration [2,5]. For this reason, it is expected that during the sensorimotor skill teaching, the expert will assist the naïve in autonomously finding the solution of the problem [6,7]. This means that the expert should encourage exploratory activity [8] and the “repetition without repetition” approach [9]. This is the repeating of the means of solving a problem, rather than repeating the solution to it (optimization by repetition). The concept of “repetition without repetition” is of particular importance in real-life tasks since we are continuously facing unique and unpredictable situations. This variability can guide one to the proper exploration of the possible solutions, increasing the learning rhythm and the knowledge generalization [10,11,12].

To assist naïve subjects in finding the correct solution for the task they are learning, there are different strategies that experts can employ during their involvement in motor teaching. The expert can provide implicit information about the task and its goals. Processing this kind of information requires the lower cognitive involvement of the naïve, allowing for the task exploration and relying on a subconscious learning process [13,14]. The expert can also provide explicit and detailed information about the task and its objectives. This approach requires high levels of cognitive processing on the part of the inexperienced individuals and increases the possibility of poor movement performance during stressful scenarios [13]. Another option is to provide guidance in the execution of the task, which can be verbal, visual, or physical [15,16]. This learning is the most specific, since the expert guides the naïve through a particular movement pattern step by step. Even if physical guidance has proven to be useful while learning motor skills, at least for a short period just after removing the assistance [17], the passive participation of the naïve can cause problems related to the absent or reduced sensory feedback, as well as the distorted relationship between sensory perception and motor responses [18]. However, approaches that encourage the appropriate sequences of movement directions have shown beneficial effects on movement structuring and sensory integration [19]. Another strategy is to segment a task into subtasks, where each subtask is learned individually [20]. The inductive transfer used in Segmentation Learning is especially efficient when the objective of the training is the generalization of the acquired knowledge, and it plays an essential role when the training data is scarce [21]. Further on, learning subtasks may enhance the understanding of the task’s requirements, enabling the adoption of explicit strategies that foster wider generalization [22].

Robotics is a viable alternative for physical guidance, and robotic systems have been used in fields such as rehabilitation [23,24], physiotherapy [25], human skill learning [26,27,28], human–human coordination [29,30], and human motor control studies [31,32,33]. In recent years, the field of sensorimotor learning has witnessed significant advancements, particularly in the use of haptic devices for enhancing learning and skill acquisition. Studies such as those by Fu and Santello [34] have explored the intricacies of force control and perception using bioinspired prosthetic hands, providing valuable insights into the integration of haptic feedback in prosthetic systems. Similarly, the efficacy of spatially separated cutaneous haptic guidance in motor skill training highlights the potential of multi-modal feedback in enhancing training outcomes in humans [35] as well as robot sensing technologies [36]. Liu et al. [37] have extended this exploration to the realm of virtual reality, examining how haptic devices facilitate the learning of dexterous object manipulation in a virtual setup. Moreover, the work by He et al. [38] on medical simulators for tissue examination training underscores the importance of multi-modal sensory feedback. Collectively, these studies contribute to a broader understanding of how haptic technologies can be effectively integrated into sensorimotor training, thus paving the way for innovative teaching methodologies in this field. However, in the field of sensorimotor learning utilizing haptic devices, an unexplored aspect appears to be the strategies for conveying instructional stimuli and related information during the learning process. In most of the existing sensorimotor skill teaching studies, the robot still acts as a demonstrator of the task that the naïve subject has to imitate [39,40,41,42,43,44,45]. All these works are of great importance and present advances in the use of robots as teachers for humans. However, the demonstrator role of the robot during the sensorimotor teaching process leaves the advantages of robot physical guidance largely unexplored.

The goal of this study is to examine teaching with robot physical guidance in a complex novel task. We present a tracking task that involves a visuomotor perturbation with continuously rotated mapping between the motor input and the resulting movement, which is aimed to generate high cognitive activity. Such cognitive activity is a consequence of the visuomotor recalibration and the creation of new motor strategies [46,47,48,49]. This research introduces a task that demands adaptation to a novel environment where conventional motor skills offer limited advantages. The task’s complexity stems from a progressively distorted connection between motor commands and their execution, featuring a continuously changing relationship between arm movements and a virtually rotated space. This type of distortion, uncommon in daily activities, challenges subjects to learn without relying on prior knowledge, emphasizing the task’s difficulty and the critical role of the teaching process in shaping performance outcomes.

We examined the proposed approach for studying sensorimotor teaching with an experiment consisting of three groups of subjects, who completed the task in three different conditions following different experimental protocols. The first group performed the experiment without haptic guidance (non-assisted learning), the second group performed the experiment with intermittent haptic guidance (assisted learning), and the third group first performed two segments of the task, followed by the main task (Segmentation Learning). At the end of the experimental protocol, we tested the ability of the subjects to generalize the acquired knowledge by randomly modifying the rotation angle of the experimental task. We aimed to determine whether a particular teaching strategy could enhance the subjects’ comprehension of the task and their proficiency in transferring this knowledge to a novel yet related scenario. We hypothesised the following:

**H1:**  *The utilization of robotic haptic guidance improves the teaching process resulting in improved learning performance for unfamiliar and challenging tasks when compared to learning without assistance.*

**H2:**  *Segmentation training setting enhances the teaching process in terms of learning performance and the generalization of unfamiliar challenging tasks compared to both assisted and non-assisted learning.*

## 2. Methods

### 2.1. Participants

A total of 27 right-handed subjects (29±5 years old, 15 males and 12 females) participated in the study. Prior to their participation, the subjects were informed about the course of the experiment and signed a written consent approved by the Slovenian National Medical Ethics Committee (No. 339/2017/7). All experimental protocols were approved by the National Medical Ethics Committee (No. 339/2017/7) and the methods were carried out in accordance with the relevant guidelines. The experimental groups were created by randomly assigning each subject to one of the three experimental groups corresponding to the three experimental conditions:

1. Non-Assisted (NAD): The subjects performed the Main Task while the robot acted merely as an interface with the virtual environment and did not intervene in the performance. The group under this condition was considered the control group.
2. Assisted (ATD): The subjects performed the Main Task and the robot assisted them by giving a guiding force. This mild force (3 N maximum) tried to guide the hand movement in the human space in order for the pointer to follow the correct path of the target in the virtual task space. The guidance can be viewed as a teaching instruction for the subjects to learn the correct movement. The trials in this condition were intermittently assisted, i.e., the assistance was given every other trial in a solo-assisted-solo sequence to track the learning process.
3. Segmentation (SEG): Subjects in this condition performed two separate sub-tasks followed by the Main Task, with the intention to maximize the generalization of the acquired knowledge. As in the ATD group, the subjects in this group also received intermittent assistance from the robot.

### 2.2. Experiment

**Figure 1:**
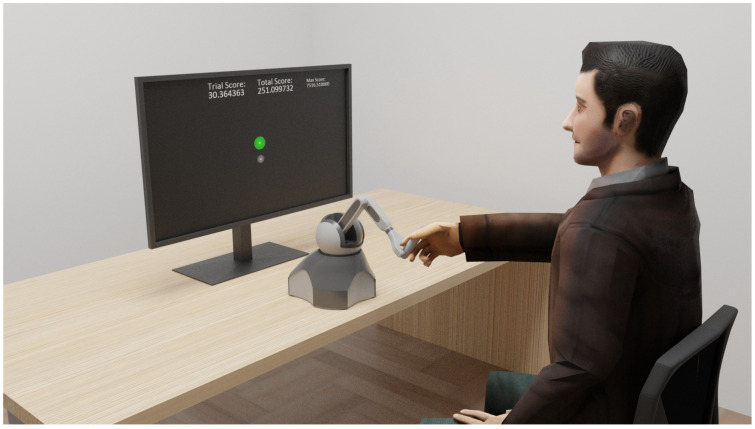
Experimental setup. The robot was aligned with the center of the screen. All subjects were requested to keep the center of their chest in line with the robot and the center of the screen.

During the experiment, the subjects were sitting in front of the screen with both feet touching the floor and without crossing their legs. They were positioned in a way such that the horizontal center of the lower screen edge, the center of the haptic robot Phantom Omni (SensAble Technologies, Wilmington, MA, USA), and the center of their chest were horizontally aligned. The manipulation of the end-effector was performed with the right hand while the left one was resting over their left leg (Figure 1).

**Figure 2:**
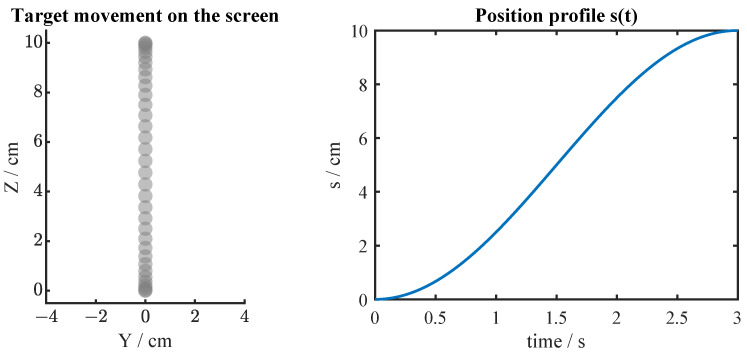
(**Left panel**): Task environment. On the screen where the virtual environment is presented, the target was moving vertically with a speed profile defined by s(t). (**Right panel**): Position change profile in the *z*-axis described by the function s(t). This profile is an approximation to the Gaussian integral curve.

The subjects had to perform a tracking task by manipulating the Phantom Omni robot (Figure 1). The robot measured the subjects’ movements and mapped them into a virtual task environment. The human uses the end-effector of the robot to control a virtual pointer, which moves in the virtual task space (Figure 2, left panel). The movements are limited to the (y,z) plane by a spring-like force acting in the *x*-axis. The mapping of the (y,z) on the screen corresponds to left and right for the *y*-axis, up and down for the *z*-axis, and in and out for the *x*-axis. Note that this mapping is only valid for when no visuomotor perturbation is in progress. The Phantom Omni robot was programmed in C++ using the CHAI3D libraries and its utilities for the creation of the virtual environment graphics.

In the virtual environment, the pointer was presented as a grey sphere with $r_{p}=0.375$ cm. The target was presented as a sphere with $r_{t}=0.5$ cm and changed among 3 colours depending on the trial stage. During the 3 s of movement the target was coloured green, letting the subjects know that they could proceed with the tracking; when the movement was completed the target changed to red, indicating the 3 s of resting; and at the end of the resting period, the target colour switched to yellow to indicate that the new trial was ready to start and the subject needed to make the pointer coincide with the target in order to start the next trial. During the assisted trials, the assistance was given by a virtual spring attached on its extremes to the target and the pointer, and with a spring coefficient $k_{p}=0.015$ N/m. Moreover, during the whole experiment, a spring force acting in the *x*-axis with a $k_{p}=0.03$ N/m helped the subjects to keep their movements in the (y,z) plane.

Subjects were instructed to align the cursor they controlled with the movement of the target. They were told the position of the cursor would not always align with the movement of their hand. They were also informed that there might be assistance from the robot on some trials. Moreover, subjects in the SEG group were told the task would change in between and that their objective was still to keep the cursor inside the target. To maintain the attention of the subjects during the experiment, we implemented a scoring system based on the accuracy of the tracking movement. The main idea behind the scoring system was to keep the subjects focused and motivated in the tracking of the target. Additionally, the use of the score served the subjects as a guide for finding the optimal movement i.e., the subject repeated and explored the movements that provided the most points. The trial score, session score, and max score across subjects were displayed on the screen throughout the whole session (Figure 1), and the subjects were instructed to aim to beat the max score.

#### 2.2.1. Experimental Task

The goal of the subjects was to track a moving target. The target moved in the z-axis and the movement followed a sinusoidal profile aimed to exhibit the response of a second-order system to reproduce the stages of a natural reaching movement: acceleration towards a target, constant velocity, and deceleration (Figure 2).

This movement is defined by the following function:

(1) $$s\left(t\right)=10\cdot \frac{\cos\left((t/t_{f})+\pi \right)+1}{2}$$

where s(t) is the movement profile function, $t_{f}$ is the time factor given by $t_{f}=t_{T}⁄\pi$, the term $t_{T}$ represents the total time duration of each trial (3 s), and *t* is the current time of the trial. The target position can be expressed with the following vector:

(2) $$p=\left[\begin{array}{c} 0\\ 0\\ s(t) \end{array}\right].$$

#### 2.2.2. Experimental Conditions

**Figure 3:**
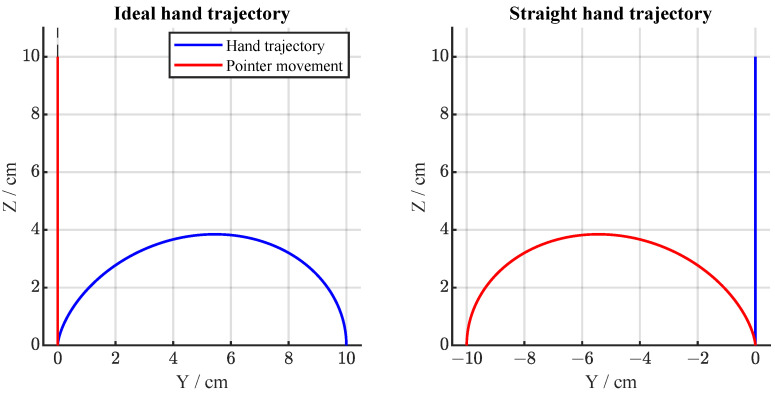
Representation of the relationship between hand movement and pointer movement during visuomotor rotation. (**Left panel**): representation of an ideal hand trajectory (blue) required to obtain a perfectly straight path of the pointer in the virtual environment (red). (**Right panel**): representation of a straight hand trajectory (blue) which would cause an arched trajectory of the pointer in the virtual environment (red).

To test the hypotheses regarding robot sensorimotor teaching, we designed a novel task setting that involves an unfamiliar visuomotor perturbation. Performing casual movements in the Cartesian space is already a familiar and intuitive motor task for humans. Thus, the objective of our experimental design is to examine the motor learning/teaching of an unfamiliar task with a high learning difficulty by itself. To facilitate this, we developed an experimental environment where the visualization of the virtual space is distorted in a way that the arm movement in either the *y*-axis or *z*-axis does not map directly to the movement of the pointer shown on the screen. This mapping followed a non-linear relationship. For example, when a subject moved straight only along the *z*-axis, the pointer in the task space would move along a curved path in the virtual task space as shown in Figure 3. This mapping was unknown to the subjects and was the goal of the motor learning/teaching process.

To study the effects of different teaching strategies and test our hypotheses, we set three experimental conditions. These conditions vary in the way the virtual environment is presented and the assistance given by the robot. For the first two experimental conditions (Assisted and Non-Assisted), we used the so-called Main Task, and for the third condition, we additionally used the so-called task segmentation.

##### Main Task

The visuomotor perturbation was emulated by inducing non-linear mapping between the movement in the human space and the actual movement of the pointer in the virtual task space. This mapping followed a rotation matrix:

(3) $$R=\left[\begin{array}{ccc} 1 & 0 & 0\\ 0 & \cos(\theta (t)) & -\sin(\theta (t))\\ 0 & \sin(\theta (t)) & \cos(\theta (t)) \end{array}\right].$$

By multiplying (2) and (3), we obtain the mapping between the movement in human space and the movement of the pointer in virtual task space:

(4) $$Rp=\left[\begin{array}{c} 0\\ -s(t)\sin(\theta (t))\\ s(t)\cos(\theta (t)) \end{array}\right],$$

where θ(t) is defined as

(5) $$\theta \left(t\right)=\phi \left(t/t_{T}\right).$$

Parameter ϕ is the maximum rotation angle to be used during the trial. The ideal hand trajectory can be imagined to be constructed by continuously rotating the initial trajectory defined with (2) by a time-dependent angle defined by (5). This mapping can be clearly understood by looking at the example hand movements presented in Figure 3.

##### Task Segmentation

The secondary tasks were designed to present the Main Task features separately, i.e., first, the subject is introduced to the rotation of the virtual reality (Rotation Task), and then is introduced to the translation part of the movement (Translation Task).

**Figure 4:**
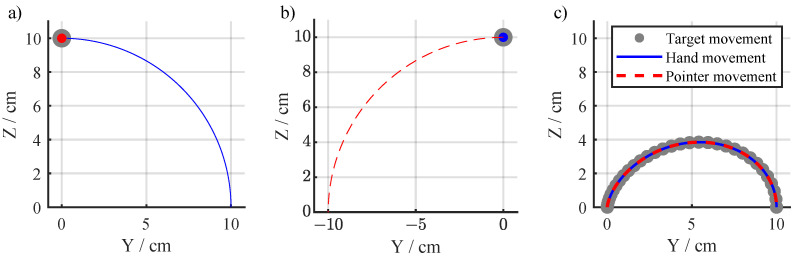
Representation of the translation and rotation tasks. (**a**) An ideal hand trajectory (blue) along the arc defined for the rotation task results in the pointer (red) remaining stationary inside the target location (gray). (**b**) During the rotation task if the hand is stationary (blue) at the starting position of (0, 10), this results in the pointer tracing an arc (red). (**c**) Representation of the Translation Task, where the target on the screen was moving along the trajectory of the Main Task (gray). The ideal hand trajectory (blue) and pointer path (red) coincide since there was no visuomotor rotation applied in this case.

Rotation Task: During the Rotation Task, the required movement to perform was a trajectory describing an arc with a 10 cm radius (Figure 4a), which can be expressed as

(6) $$p_{RT}=\left[\begin{array}{c} 0\\ 10\sin(\theta (t))\\ 10\cos(\theta (t)) \end{array}\right].$$

As a consequence of the combination of the target movement and the frame’s rotation, the target is shown on the screen as apparently static at the top of the screen. However, if the subjects do not make any movement, the visual effect will be that the pointer moves away from the target towards the left with an arched trajectory (Figure 4b). To solve the task correctly, the subject has to move the end-effector of the robot towards the right in order to keep the pointer overlapped with the target (Figure 4a). The rationale of this secondary task is that by experiencing this kind of perturbation, the subjects can learn to adapt their movements to the virtual rotation. This would guide them to the understanding that the environment perceived visually does not correspond to “normal” visuomotor proprioception.

Translation Task: In the Translation Task, the required movement is given by (4) (Figure 4c), without any rotation of the virtual task space. With this, the subject is able to see the real trajectory followed by the target. This subtask is considered the easiest and it is intended for the subjects to explore the movement they need to perform to complete the Main Task successfully.

### 2.3. Experimental Protocol

**Figure 5:**
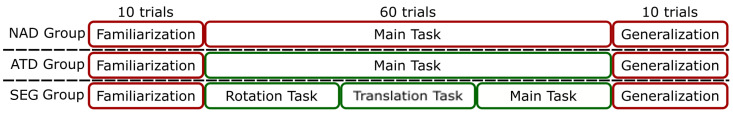
Experimental protocol followed by the different groups. The red squares represent the non-assisted stages, and the green squares represent the intermittently assisted stages.

The experiment consisted of three sessions with a separation period of a minimum of 12 and a maximum of 48 h between them. In each session, the subjects had to complete a total of 80 trials (Figure 5). Each trial consisted of 3 s of target tracking, 3 s of rest, and a standby period the length of which was decided by the subject. The sessions were divided into three main stages:

1. Ten Familiarization trials: The ϕ angle was set at 0 degrees. This stage was used to let the subjects familiarize themselves with the virtual environment during the first session, and as memory washout during the second and third sessions.
2. Sixty Training trials: The ϕ angle was set at 90 degrees. This stage determined the difference among groups:
  (a) NAD group performed 60 Main Task trials unassisted.
  (b) ATD group performed 60 Main Task trials with intermittent assistance.
  (c) SEG group performed 20 Rotation Task trials followed by 20 Translation Task trials and 20 Main Task trials, intermittently assisted.
3. Ten Generalization trials: During this stage, the subjects were presented with a version of the Main Task where the ϕ angle changed in a pseudo-random sequence from trial to trial with possible values between 0 and 90 degrees in intervals of 10 degrees (30, 0, 60, 40, 10, 80, 50, 90, 20).

### 2.4. Data Analysis

**Figure 6:**
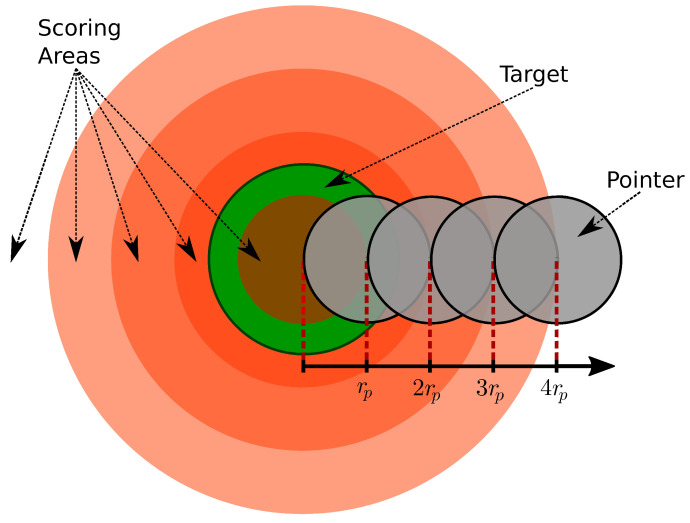
Visual representation of the scoring system. The scoring areas (orange) are concentric to the target (green). The scoring areas are the zones of the virtual space where the subjects can collect accuracy points the radii of which are related to the pointer radius $\left(r_{p}\right)$. The amount of points collected is dependent on the distance factor presented in (7), which is calculated by measuring the Euclidean distance between the centers of the target and the pointer. The grey circles representing the pointer are situated at the outer edge of the scoring areas.

To observe motor learning, we evaluated the accuracy of the movements. The accuracy was measured with respect to the radius of the pointer $\left(r_{p}\right)$. To calculate the sum of given points, we set 5 scoring areas around the target (Figure 6), where each area differed by 25% of the total amount of points with the areas around it. The percentage of points acquired was saved in the distance factor $d_{f}$, which was defined as the weighted Euclidean distance given by the following equation:

(7) $$d_{f}=\left\{\begin{array}{c} 1\mathrm{when}d\leq r_{p}\\ 0.75\mathrm{when}r_{p}<d\leq 2r_{p}\\ 0.5\mathrm{when}2r_{p}<d\leq 3r_{p}\\ 0.25\mathrm{when}3r_{p}<d\leq 4r_{p}\\ 0\mathrm{otherwise} \end{array}\right.$$

where *d* is the Euclidean distance from the center of the target to the center of the pointer. The score was dependent on the amount of time spent in each one of the scoring areas and it is calculated at the end of every haptic cycle (1 kHz). The result was added to the accumulated score of the trial; thus, the score was defined as

(8) $$score=100\cdot \frac{\sum \left(t_{i}-t_{i-1}\right)d_{f_{i}}}{t_{T}}$$

where the expression $\left(t_{i}-t_{i-1}\right)$ is the period of time being evaluated and compensates for the variability of the haptic cycle (1 kHz ± 5 Hz) due to the system delays. With these metrics, subjects can reach a maximum of 100 points per trial and 8000 points per session.

We additionally looked at the max error during learning. Max error was determined as a maximum Euclidean distance from the center of the target to the center of the pointer during each trial.

To properly look into the learning process, we decided not to consider the even trials (in which the robot assistance was present) in the analysis. Neither did we consider the trials in which the SEG group was changing conditions, since these trials presented errors unrelated to the task performance but to the unexpected condition change.

To be able to observe the learning process, several subjects were discarded due to an outlier performance, and we kept acquiring data until we obtained three balanced groups in the number of subjects who successfully showed the learning of the task. The outliers’ performance was determined by calculating the mean and standard deviation (σ) at the last training trial and all the subjects that fell out of the 3σ from the mean were discarded. To understand the distribution of the discarded subjects, three of them belonged to the NAD group, one belonged to the ATD, and one to the SEG group.

To perform the statistical analysis and explore the statistical significance of the results among the different experimental groups, we used the one-way ANOVA. We set the statistical significance of the analysis as alpha=0.05. Further, we performed the post-hoc *t*-tests with Bonferroni correction. ANOVA, *t*-tests, and Bonferroni correction analysis were performed in R. The data points are reported as a mean ± standard error unless specified otherwise.

## 3. Results

The results of the performance analysis can be divided into two sections: the training and generalization phases. In the training phase, we focused on the performance evolution during three sessions of the experiment, which denotes the learning process experienced by the subjects. In the generalization phase, we analyzed the ability to transfer the acquired knowledge into scenarios similar to the one learned during the training phase.

### 3.1. Training Phase

**Figure 7:**
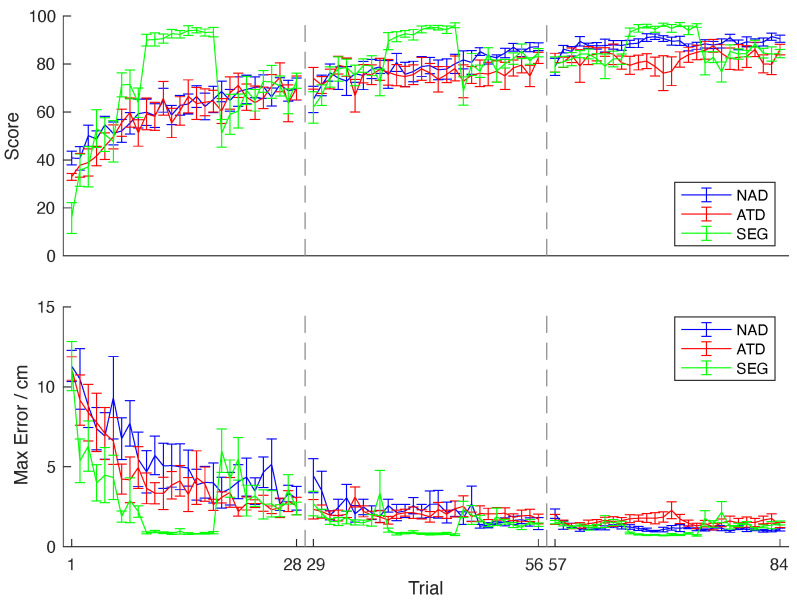
Training scores and max errors of the experimental groups: The graphic shows scores and max errors for the non-assisted trials of the experimental protocol. The Non-Assisted group, which is the control group, is presented in blue (NAD), Assisted group in red (ATD), and Segmentation group in green (SEG). The dashed grey lines divide each one of the sessions, and the standard error is represented by the error bars. Each session only includes odd trials in which the robot assistance is not present without 2 trials where the conditions were changing.

The subjects in each group performed three sessions of the experiment (Figure 7). The NAD group had no assistance during training, the ATD group had intermittent assistance, and the SEG group first completed the Rotation and Translation Tasks, followed by the Main Task with intermittent assistance. To compare learning between the NAD, ATD, and SEG groups during the Main Task, we looked at the average scores and max errors for the trials when all the groups performed the Main Task without assistance. The average scores and max errors for all the groups across the sessions are presented in Table 1. There was no significant difference in the average score (F(2,26)=0.03,p= 0.975) or average max error (F(2,26)=0.90,p= 0.420) between the groups for the first session.

However, the subjects of the SEG group were exposed to the Main Task only at the end of the first session (trial 19 of Figure 7), after they completed the Rotation and Translation Tasks. The average score of the SEG group on their first exposure to the Main Task was 51.05±5.78, which is on average 10 points higher than the scores of the NAD group (40.77±2.89) and 19 points higher than the ATD group (32.93±1.45) (trial m top panel of Figure 7 top). The ANOVA test showed the difference among the groups was statistically significant (F(2,26)=5.64,p< 0.01). The post hoc t-tests with Bonferroni correction showed that only the difference between the ATD and SEG groups was significant (t(2,16)=3.04,p= 0.016). The average max error of the SEG group on their first exposure to the Main Task was 6.01±4.08 cm, which is lower than the max error of the NAD group (11.30±2.91) and the ATD group (11.15±2.20) cm (trial 1 on bottom panel of Figure 7). The ANOVA test showed the difference among the groups was statistically significant (F(2,26)=8.17,p= 0.002). The post hoc t-tests with Bonferroni correction showed a significant difference between the NAD and SEG groups (*t*(2,16) = 3.32, *p* = 0.009) as well as the ATD and SEG groups (t(2,16)=3.17,p= 0.012).

There was no significant difference in the average score (F(2,26)=2.80,p= 0.081) or average max error (F(2,26)=1.07,p= 0.360) between the groups for the second session. There was also no significant difference in the average score (F(2,26)=2.36,p= 0.116) or average max error (F(2,26)=2.78,p= 0.082) between the groups for the third session.

All the groups improved their scores throughout the sessions, as seen in Table 1. There was a significant difference between the first and the last session for all three groups (t(2,16)=4.72,p< 0.001, t(2,16)=2.54,p= 0.022, t(2,16)=3.88,p= 0.001 for the NAD, ATD, and SEG groups, respectively). There was also an improvement throughout the sessions in terms of the max errors. There was a significant difference between the first and the last session for all three groups (t(2,16)=3.81,p< 0.002, t(2,16)=2.39,p= 0.029, t(2,16)=2.73,p= 0.015 for the NAD, ATD, and SEG groups, respectively).

While the NAD and ATD groups only performed the Main Task, the SEG group also performed the Rotation and Translation Tasks. In the Rotation Task, the accuracy improved throughout the experiment, with the scores going from an average of 49.62±13.03 in the first session to 74.01±9.55 at the second session to 81.83±5.01 at the last session (*F*(2,26) = 26.65, p< 0.001), while the max errors decreased from 4.75±2.23 to 1.98±0.83 cm to 1.40±0.23 cm in the last session (F(2,26)=15.14,p< 0.001). The Translation Task was performed with relatively similar efficiency throughout the experiment, with the scores going from an average of 92.24±4.64 in the first session to 93.43±3.46 to 95.11±2.37 at the last session (*F*(2,26) = 1.44, p= 0.256), while the max errors went from 0.87±0.13 to 0.85±0.13 cm to 0.76±0.09 cm in the last session (F(2,26)=2.14,p= 0.139).

### 3.2. Generalization

**Figure 8:**
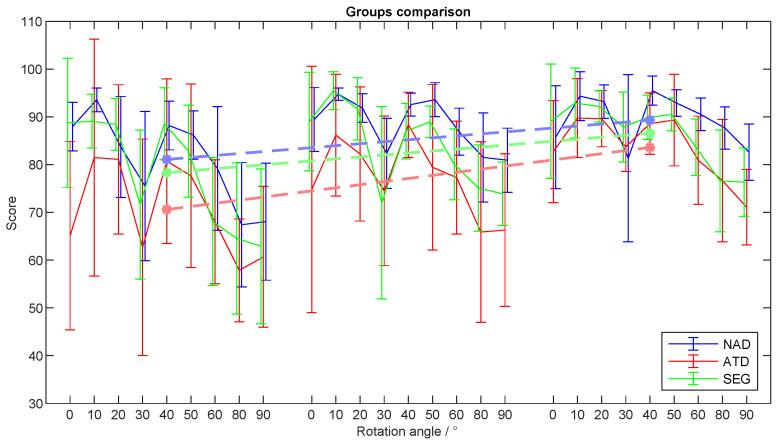
Scores from the generalization phase of the experiment. The rotation angles during the familiarization phase are set in a pseudorandom sequence. In the figure, the rotation angles are arranged in ascending order to facilitate data interpretation. The Non-Assisted group, which is the control group, is presented in blue (NAD), the Assisted group in red (ATD), and the Segmentation group in green (SEG). The standard error is represented by the error bars.

To test if different teaching strategies influence one’s capacity to apply and transfer acquired knowledge to related tasks, we examined the performance during the generalization phase. The evaluation of the teaching strategies used during the generalization phase was performed by analyzing the subjects’ performance during the trials in which the rotation angle θ changed from trial to trial. In Figure 8, the values of θ are organized in ascending order to facilitate data interpretation.

The average scores and max errors for all the groups across the sessions are presented in Table 2. There was no significant difference in the average score (*F*(2,26) = 2.85, p= 0.077) or average max error (F(2,26)=4.09,p= 0.029, post hoc tests for NAD-ATD, NAD-SEG, and ATD-SEG comparisons: t(2,16)=2.37,p= 0.080, t(2,16)=0.34,p=1.0, t(2,16)=2.01,p= 0.123) between the groups for the first session.

The scores for the NAD group in the second session were statistically higher compared to the ATD and SEG groups (F(2,26)=5.18,p= 0.013; t(2,16)=2.77,p= 0.027, t(2,16)=2.63,p= 0.037). Looking at the average max errors in the second session, the statistical analysis revealed a difference in the max errors between the NAD and ATD groups (F(2,26)=5.22,p= 0.013, t(2,16)=2.66,p= 0.034). There was no significant difference in the average score (F(2,26)=3.23,p= 0.057) or average max error (F(2,26)=2.25,p= 0.127) between the groups for the third session.

The average generalization performance improved from the first to the last session, as can be seen in Table 2. The improvement was statistically significant for all three groups (t(2,16)=3.52,p= 0.003, t(2,16)=2.57,p= 0.021, t(2,16)=2.99,p= 0.009). Similarly, the max errors improved from the first to the last session. The improvement was statistically significant for all three groups (t(2,16)=2.94,p= 0.010, t(2,16)=2.40,p= 0.029, t(2,16)=2.57,p= 0.021).

The generalization scores of all the groups looked inversely proportional to the rotation angle; the greater the rotation angle, the lower the score achieved (Figure 8). With the exception of the $30^{{}^{\circ}}$ rotation angle, the average scores in all the groups when the rotation angle was in the range of 0–50° maintained a value around 90 points. The average scores at a $30^{{}^{\circ}}$ rotation showed an increase session after session. Similarly, the scores when the rotation angle was $60^{{}^{\circ}}$, $80^{{}^{\circ}}$, and $90^{{}^{\circ}}$ showed an increase throughout the experiment.

## 4. Discussion

During the expert–naïve interaction in sensorimotor teaching, the stimuli from the teaching expert are often influenced by the previous knowledge of the naïve [21], which can either potentiate or bias the learning towards pre-existing skills. This study introduces a task designed to exploit a new environment where prior motor knowledge is minimally useful. The task involves a constantly changing mapping from arm movement to a virtually rotated space, a requirement not typically found in daily activities.

The intricacy of our tasks lies in the progressively altered correlation between motor commands and their execution. This type of progressive distortion is atypical in everyday life, thereby hindering subjects from drawing on prior knowledge. The inability to utilize existing knowledge renders the task challenging to master without instructional intervention, leading to the assumption that the observed performance outcomes are intrinsically linked to the teaching methodology employed. Relevant studies on fabric-based haptic devices for motor learning and on enhancing fingertip force learning through haptic feedback support the significance of specialized teaching approaches in learning novel motor tasks [50,51].

The task was used to evaluate the effects of different sensorimotor teaching techniques through haptic guidance, where we explored the use of Assisted Learning and Segmentation Learning in the training of human subjects. The assisted teaching technique used was limited to a spring-like haptic assistance. The two groups in which such techniques were applied were compared against a control group where the subjects solved the task without any assistance. All the groups were capable of learning the task along the three sessions of the experiment, which showed that the designed experimental task with visuomotor perturbation was learnable to begin with. All the groups improved their scores and decreased their errors from the first to the last session. However, there were a few subjects that were not able to successfully learn the task and were discarded. Three of them belonged to the NAD group, one belonged to the ATD, and one to the SEG group.

On average, the NAD, ATD, and SEG groups had a similar performance during the training. Contrary to what was expected, the Main Task scores were practically equal between the groups at the end of all the sessions. Moreover, the NAD, ATD, and ATD groups showed a similar performance in their errors with comparable errors during the first, second, and third sessions. The performance scores and errors of the SEG and ATD groups, which were assisted during learning, do not show any observable advantages, which goes against hypothesis H1. Moreover, the performance scores and errors of the SEG group do not show any observable advantage on the use of the segmentation training, which goes against hypothesis H2.

The subjects of the SEG group were exposed to the Main Task only at the third stage of the first session following the Rotation Task and Translation Task stages. Their average scores showed a better performance than the other two groups when they were exposed to the Main Task for the first time. The maximum error values confirmed the better performance of the SEG group on their first exposition to the Main Task which can be attributed to the influence of the rotation and translation subtasks. This was expected since the Translation Task stage included the same physical movement of the arm as in the later Main Task; therefore, the subjects were only required to maintain that movement but with different visual stimuli. This shows how learning a specific physical movement first can have a positive influence on the initial performance in a more complicated task.

In the generalization phase, the performance of the groups was comparable during the first session. However, during the second session, the performance of the NAD group was slightly better than the performance of the ATD and SEG groups. In the third session, the performance of all the groups was again comparable with no statistical differences in the scores and errors between the groups. The lack of a significantly better performance of the SEG group is attributed to the fact that the multitask technique requires longer periods of training in order to show its advantages as well as the careful planning of the task sequence presented to the subjects [52,53].

The planning of the task sequence is a key factor in the achievement of the “repetition without repetition” training approach. This approach, often underscored by the inductive transfer inherent in Segmentation Learning, becomes particularly advantageous when the training objective leans towards enhancing the generalization of learned knowledge. Immersing learners in subtasks can also amplify their grasp of the comprehensive task requirements and lead to the application of explicit strategies in the generalization process. According to hypothesis H2, the effects of this training approach were expected to manifest in the performance of the SEG group during the generalization phase. The rationale was that the participants within this group would develop enhanced a comprehension of the task and proficiency in transferring this knowledge to novel yet related scenarios. However, contrary to what we expected, the subjects in the SEG group did not have a better generalization performance than their counterparts in the ATD and NAD groups.

Previous studies have shown that the learning of rotations up to $90^{{}^{\circ}}$ is exponential and shows limited generalization [49]. The main difference with these studies is that the rotation in our study was continuous in time during the whole trial instead of fixed. Since the order of the rotation degrees in the generalization phase was fixed in our experiment, the performance in each trial might have been heavily influenced by the rotation amplitude of the preceding trial. However, especially the scores beyond $60^{{}^{\circ}}$ improved significantly from the first to the last session. This suggests that with further training, the performance decay will disappear and the scores achieved at all the rotation angles will have similar values despite being exposed only to $0^{{}^{\circ}}$ and $90^{{}^{\circ}}$ rotation angles.

The results of the experiment did not prove our initial hypotheses, which might be due to some of the limitations of our study. Human–robot interaction during sensorimotor teaching is complicated due to the complex interaction between the human, the robot, and the environment. As a consequence, knowledge acquisition is not merely determined by prior information and skills [28,37]. There was only haptic assistance without any other visual or audio feedback during the intermittent assistance phase. Perhaps additional visual or audio stimuli could better reinforce the learning of the complex mapping between the hand and pointer movement [4]. Additionally, in order to increase the sense of the immersion of the subjects, the experimental setup could be upgraded to use stereo vision using a VR headset. Hardware limitations, i.e., the small force applied for the assistance could also be the reason for the lower scoring of the ATD group. Additionally, the segmentation approach for the task might have been nonintuitive for the subjects, so exploring additional tasks with various ways of segmentation could prove to be more effective. Moreover, as we mentioned before, the task is designed to avoid interference with daily living tasks. However, the concept of daily living tasks is a constantly evolving concept that mutates together with society and the technology available to the general public. For this reason, it is important to consider that modern society has extensive exposure to virtual environments and this influences the results of using robotic devices and virtual agents for sensorimotor teaching. This would be in line with some studies that report a superior real-to-virtual environment mapping in gaming subjects with respect to those who normally do not have contact with video games [54,55].

In future work, we will aim to verify the observations about how the gaming experience can influence the results of various visuomotor performances. Another very important aspect of future experimentation would be to explore the individual responses to the teaching strategy. Based on these responses, the teaching strategy could be adapted, taking into consideration contextual factors and the individual motor skills in order to provide effective teaching with the long-term retention of these skills. The individualization of teaching strategies has been proven to be useful when the training is directed to the performance of a specific task [56], but the question of how to improve the generalization of the acquired knowledge and how to ensure the subjects’ long-term retention of these skills remains open. The inclusion of sensorimotor teaching techniques can give a new direction to the robot assisted motor learning. Nevertheless, the robots must include robust motor teaching procedures in their algorithms in order to be able to act as sensorimotor teachers. This requires the creation of faithful models of teaching and learning processes in mathematical formulations so that they can be used by the robot control systems. The mathematical models developed in the area of motor learning have been mainly directed to the kinematic and muscle actuation of the movement and the movement primitive approach [57,58,59,60,61,62]. In the implementation of these learning models, the concepts of kinesthetic teaching are a common feature [53], and several conceptual models of the expert-naïve relationship have been presented [63,64,65], but the mathematical formalization of such concepts have lacked proper attention.

Overall, our results indicate that the task design is useful for the study of sensorimotor teaching and for the generalization of the knowledge acquired in the process. The metrics presented allow us to explore the evolution of accuracy and precision during the training sessions.

## Figures and Tables

**Table 1:** Average training scores and max errors of the experimental groups across sessions.

	NAD		ATD		SEG	
	**Score**	**Error /cm**	**Score**	**Error/cm**	**Score**	**Error/cm**
Session 1	68.84 ± 12.54	3.81 ± 2.15	68.19 ± 15.25	2.65 ± 1.26	67.42 ± 12.16	3.27 ± 2.00
Session 2	84.69 ± 4.47	1.63 ± 0.56	77.19 ± 9.64	1.96 ± 0.85	81.29 ± 4.77	1.59 ± 0.33
Session 3	89.41 ± 3.70	1.08 ± 0.18	83.57 ± 9.82	1.52 ± 0.65	83.86 ± 3.67	1.43 ± 0.27

**Table 2:** Average generalization scores and max errors of the experimental groups across sessions.

	NAD		ATD		SEG	
	**Score**	**Error/cm**	**Score**	**Error/cm**	**Score**	**Error/cm**
Session 1	81.08 ± 5.84	0.18 ± 0.05	70.59 ± 13.94	0.33 ± 0.19	78.31 ± 7.16	0.19 ± 0.07
Session 2	88.20 ± 3.02	0.12 ± 0.03	77.21 ± 11.50	0.22 ± 0.12	83.68 ± 4.19	0.15 ± 0.04
Session 3	89.31 ± 3.87	0.11 ± 0.03	83.59 ± 6.03	0.16 ± 0.08	86.55 ± 4.12	0.12 ± 0.02

## Data Availability

The raw data supporting the conclusions of this article will be made available by the authors on request.
